# The Association of Perceived Sustainable and Informatics Leadership with Employee Happiness Among Nurses: A Cross-Sectional Study †

**DOI:** 10.3390/healthcare14213804

**Published:** 2026-11-06

**Authors:** Burcu Genç Köse, Ayşe Gümüşler Başaran, Bahar Kefeli Çol

**Affiliations:** 1 Faculty of Health Sciences, Recep Tayyip Erdoğan University, Rize 53020, Turkey; 2 Güneysu School of Physical Therapy and Rehabilitation, Recep Tayyip Erdoğan University, Rize 53350, Turkey

**Keywords:** happiness, sustainability, nurse, hospital, leadership, informatics

## Abstract

**Background/Objectives:** In technology-driven healthcare, examining the relationship between nurses’ happiness and their perceptions of sustainable and informatics leadership is important. This study examined the relationship between nurses’ perceptions of sustainable leadership and informatics leadership and their levels of employee happiness. **Methods:** This was an analytical cross-sectional study conducted at a Training and Research Hospital and a State Hospital in one province. The study included 158 nurses who agreed to participate. A nurse information form, the Sustainable Leadership Scale, the Informatics Leadership Scale, and the Employee Happiness Scale were used to collect the data. **Results:** Nurses’ perceived sustainable leadership score was 35.49 ± 11.37, their perceived informatics leadership score was 56.93 ± 18.19, and their employee happiness score was 58.18 ± 14.49. Positive correlations were found between perceptions of sustainable leadership and perceptions of informatics leadership, between perceptions of sustainable leadership and employee happiness, and between perceptions of informatics leadership and employee happiness. Multiple linear regression analysis showed that sustainable leadership (B = 0.523, 95% CI: 0.263–0.783) and informatics leadership (B = 0.180, 95% CI: 0.025–0.335) were positively associated with employee happiness after adjustment for demographic and occupational characteristics. **Conclusions:** Both sustainable leadership and informatics leadership perceptions were positively associated with nurses’ employee happiness scores. These findings suggest that leadership perceptions may be relevant to employee happiness in nursing settings. Healthcare institutions may consider promoting employee participation in decision-making, prioritizing staff well-being, and providing professional development opportunities related to sustainable and informatics leadership.

## 1. Background

The concept of sustainability, which has taken shape in response to new needs arising from an evolving world, has become a significant topic of interest today. Defined as the ability to endure over time, sustainability represents an approach aimed at ensuring that relationships across various fields are conducted in harmony with one another [1]. Economic and social crises, particularly in developing countries, have made the importance of the sustainability process in the healthcare sector even more apparent [2]. Adopting the concept of sustainability in the delivery of nursing services will significantly contribute to the sustainability of the healthcare sector [3]. In this context, adopting a sustainability framework in nursing services—one of the fundamental elements supporting the sustainability of healthcare systems—is of great importance.

Sustainable leadership advocates for the ability to make long-term decisions, foster innovation within the organization, and build a team loyal to the institution. Sustainable leadership, which aims to balance an organization’s life cycle and human resources, champions the delivery of high-quality services [4,5]. It is an approach that enhances organizational success [6]. To establish sustainability, organizations must adopt and implement the philosophy of sustainable leadership [7]. In the healthcare sector, the concept of sustainable leadership among nurse managers—who play a major role in the continuous provision of healthcare services, the retention of qualified staff, and the planning of service delivery that is responsive to patient needs—is of great importance [8,9].

Leadership style in nursing positively influences employee job satisfaction [10], and in this regard, sustainable leadership can contribute to organizational success. It can also encourage employees to think innovatively and foster a supportive atmosphere [4]. The sustainable leadership approach can also positively influence employees’ well-being [5,11]. Leaders who adopt a sustainable leadership approach can, in accordance with ethical standards [12], foster trust among employees, cultivate a culture of integrity and accountability, and positively influence employee satisfaction [4].

In addition to ensuring the organization and coordination of their teams, nurse leaders may face numerous social and psychological challenges in delivering healthcare [13]. While the goals nurse leaders aim to achieve at the organizational level remain unchanged, the environments they use to achieve them may vary in today’s technological landscape [14]. In particular, the rapid advancement of informatics technology is creating new fields [15]. Developments in technology play a significant role in improving the quality of healthcare and nursing services [16]. In this context, the emergence of informatics leadership has influenced leaders’ management styles [15].

Informatics leadership is defined as the ability to influence groups and organizations to enhance communication and facilitate the use of information [17]. It involves recognizing group members’ needs and interests and encouraging them to work together to achieve individual or organizational goals [15]. In nursing, informatics technology involves using and managing information processes to support patients and staff in the decision-making process [18]. Informatics leadership is a leadership style that supports information sharing within organizations and enhances collaboration [19]. A nursing informatics leader is an individual skilled in anticipating and planning for potential future improvements and changes [20]. In one study, 61.9% of nurse managers reported feeling a lack of training in informatics leadership [21]. Informatics leadership that encourages information sharing strengthens employees’ sense of control and positively influences their performance [22]. Furthermore, leadership behaviors that support knowledge sharing are associated with employees’ creative problem-solving capacity and creative performance [23].

Happiness, a key factor in protecting an individual’s health, is an emotion expected of all employees. It is particularly needed among nurses, from whom behaviors such as dedication, self-confidence, and energy are expected [24]. Studies have found a positive relationship between nurses’ happiness and work–life balance [25], and it has been determined that the leadership traits desired by employees [26,27] are positively associated with nurses’ happiness levels. Leadership style appears to be a significant organizational factor associated with employee happiness. Previous research has examined the relationship between sustainable leadership and workplace happiness among healthcare employees. However, the rapid advancement of information technologies and the increasing digitalization of information management and communication processes in healthcare have heightened the importance of informatics leadership. In this context, understanding how informatics leadership practices are associated with employees’ perceptions and levels of happiness has become increasingly important. Accordingly, examining informatics leadership alongside sustainable leadership allows the independent associations of both leadership approaches with employee happiness to be evaluated, potentially providing a broader perspective on their relationships with employee happiness among nurses and contributing to a more comprehensive understanding of these associations in the existing literature.

In the context of healthcare delivery, where technology plays an important role and continuity of care is of critical importance, this study examines the relationship between the two leadership approaches perceived by nurses and their levels of employee happiness, specifically seeking to answer the following research questions: (1) What are nurses’ levels of perceived sustainable leadership, perceived informatics leadership, and employee happiness? (2) Are nurses’ perceptions of sustainable leadership and informatics leadership associated with their levels of happiness? (3) To what extent do nurses’ perceptions of sustainable leadership and informatics leadership predict employee happiness? The findings may inform improvement strategies in leadership development initiatives by highlighting how sustainable and informatics leadership approaches are associated with employee happiness. The findings may contribute to the literature by providing evidence on the associations of both leadership approaches with employee happiness in nursing settings.

## 2. Material and Methods

### 2.1. Objective

The purpose was to examine the relationship between nurses’ perceptions of sustainable leadership and informatics leadership and their levels of employee happiness.

### 2.2. Study Population and Sample

This study was designed as an analytical cross-sectional study. The study population consisted of 400 nurses actively working at a Training and Research Hospital (250 nurses) and a State Hospital (150 nurses) in a province. Nurses whose positions were registered at these hospitals but who were assigned to other institutions or were on leave during the data collection period were not included in the study population. Instead of selecting a sample, the aim was to reach the entire population. The study was completed with 158 nurses, corresponding to a response rate of 39.5%. All 400 eligible nurses were approached for participation. The remaining 242 nurses declined to participate, stating that they did not wish to complete the questionnaires. No eligible nurses were unreachable, and no questionnaires were excluded due to incomplete responses. The inclusion criteria were actively working as a nurse at one of the participating hospitals and voluntarily agreeing to participate in the study. Questionnaires were not administered to nurses who declined to participate. The 19 participants identified as managers in the study were unit/ward nurse managers. While the leadership scales were being completed, all participants were asked to evaluate senior management (the Directorate of Health Care Services). Therefore, all participants evaluated the leadership practices of their senior managers, and no leadership self-assessment was conducted. Data were collected between April and June 2026.

### 2.3. Data Collection Tools

Data were collected using a nurse information form, the Sustainable Leadership Scale, the Informatics Leadership Scale, and the Employee Happiness Scale. The researchers visited all units of both hospitals during working hours and distributed the questionnaires directly to nurses. Participants were given approximately two weeks to complete the questionnaires, and the recruitment process continued until all eligible nurses had been approached. To ensure anonymity, the questionnaires were collected without personal identifying information. Senior management was not involved in the distribution or collection of questionnaires and did not directly monitor the data collection process, thereby minimizing the possibility of identifying individual participants or their responses.

- Nurse Information Form: The form consisted of a total of 11 questions: 6 questions about the nurse’s age, gender, employer, educational status, perceived income level and marital status, and 5 questions regarding their position, years of professional experience, years of service at the institution, weekly working hours, and work schedule.
- Sustainable Leadership Scale: Developed by Dalati et al. (2017), this scale assesses perceived sustainable leadership behaviors [28]. Zorlu and Korkmaz (2020) [29] adapted the scale into Turkish and assessed its validity and reliability. Exploratory factor analysis supported the single-factor structure, explaining 69% of the total variance, while confirmatory factor analysis demonstrated good model fit. The average variance extracted (AVE) was 0.654, and composite reliability (CR) was 0.950. These findings provide evidence of the scale’s construct validity and reliability in the Turkish context. The scale’s validation study indicated that it was compatible with the Turkish management system [29]. Consisting of 10 items and a single dimension, the scale uses a 5-point Likert scale. Responses are rated on a scale ranging from (1) “Strongly Disagree” to (5) “Strongly Agree.” The scale contains no reverse-scored items. A higher scale score indicates a higher level of sustainable leadership. In the study adapting the scale to Turkish, Cronbach’s alpha (α) was 0.930. In this study, Cronbach’s alpha (α) was 0.981.
- Informatics Leadership Scale: Developed by Ulutaş and Arslan (2018) [17], this scale—which has undergone validity and reliability testing—measures perceptions related to informatics leadership. The original validation study reported that the scale explained 79.315% of the total variance. Confirmatory factor analysis also provided evidence supporting its factor structure. The original validation study indicated that the scale was suitable for use in different fields [17]. The Informatics Leadership Scale consists of 18 items rated on a 5-point Likert scale, ranging from (1) “Strongly Disagree” to (5) “Strongly Agree.” The scale comprises three subscales: Direction, Communication, and Knowledge. These dimensions address perceived leadership behaviors related to guiding employees in the use of information technologies, using technological tools in organizational communication and management processes, and following developments in informatics. In the present study, the scale was used to assess nurses’ perceptions of their senior managers’ informatics-related leadership behaviors rather than to directly measure their clinical health informatics competencies. Although the scale comprises three subscales, the total score was used to represent overall perceived informatics leadership, and the subscales were not analyzed separately. The theoretical total score ranges from 18 to 90, while the mean item score ranges from 1 to 5. The scale contains no reverse-scored items. A higher score indicates a stronger perception of informatics leadership. In the original study, Cronbach’s alpha (α) was 0.97. In this study, Cronbach’s alpha (α) was 0.980.
- Employee Happiness Scale: Developed by Akduman (2021), this five-point Likert-type scale has undergone validity and reliability testing [30]. The scale is scored using response options ranging from (1) “I strongly disagree” to (5) “I strongly agree.” It consists of 20 items. It has five subscales: managers, coworkers, working conditions, opportunities for self-actualization, and recognition and rewards. Possible scores range from 20 to 100. The scale has no reverse-coded items. Scores below 60 indicate employee unhappiness [30]. In the original study of the scale, Cronbach’s alpha (α) was 0.89. In this study, Cronbach’s alpha (α) was 0.907.

### 2.4. Data Analysis

The SPSS 29.0 software package (IBM Corp., Armonk, NY, USA) was used for statistical analysis. Descriptive statistics were expressed as percentages, means, and standard deviations. Data were considered to follow a normal distribution if the skewness coefficient fell within the range of −1.00 to +1.00 [31]. Student’s *t*-test, ANOVA and the Bonferroni post hoc test, the Pearson correlation analysis, and linear regression analysis were used in the data analysis. In the correlation analysis, a correlation coefficient of 0–0.39 was considered a weak relationship, 0.40–0.69 a moderate relationship, 0.70–0.89 a strong relationship, and 0.90–1.00 a very strong relationship.

### 2.5. Ethical Considerations

The research was conducted in accordance with the Declaration of Helsinki and the ethical principles of the relevant university’s ethics committee between 6 April and 30 June 2026. The study was conducted after obtaining ethical approval from the relevant university’s Social and Humanities Ethics Committee (18 February 2026/69) and institutional approval from the Provincial Health Directorate (3 April 2026/6198). Since research involving human participants requires the protection of individual rights, the “Informed Consent” form and the principle of voluntariness served as the basis for the study. The “Principle of Confidentiality” was upheld by informing the nurses participating in the study that their information would not be disclosed to others. Permission to use the scales was obtained from the individuals who had previously conducted the validity and reliability studies in Turkish.

## 3. Results

Participant demographics showed that 85.4% were women, with a mean age of 33.91 ± 9.15. Of the nurses, 60.8% worked at a Training and Research Hospital, and 39.2% worked at a State Hospital. Additionally, 82.3% held a bachelor’s degree or higher, 47.5% reported that their income was equal to their expenses, 53.2% were married, and 88% held the position of ward nurse. The percentage of nurses with 10 years or less of professional experience was 60.8%, and the percentage with 10 years or less of service at their current institution was 76.6%. It was determined that 78.5% of nurses worked 41 h or more per week, and 71.5% worked both night and day shifts (Table 1).

The mean perceived sustainable leadership score was 35.49 ± 11.37, exceeding the theoretical midpoint of 30 (possible range: 10–50). Similarly, the mean perceived informatics leadership score was 56.93 ± 18.19, slightly exceeding the theoretical midpoint of 54 (possible range: 18–90). The mean employee happiness score was 58.18 ± 14.49, which was below the cutoff score of 60 reported in the original scale development study (Table 2). Only 3 participants (1.9%) scored 60 points, while 55.7% scored below 60 and 42.4% scored above 60. Of the 158 participants, 88 (55.7%; 95% CI: 48.0–63.2%) had employee happiness scores below 60.

Nurses’ mean perceptions of sustainable leadership and informatics leadership were above their respective theoretical midpoints, whereas their mean employee happiness score was below the specified cutoff value.

In the analysis of Employee Happiness Scale scores according to the independent variables, significant differences were found by employer, position, weekly working hours, and perceived income level (respectively *p* < 0.001, *p* = 0.009, *p* = 0.046, and *p* = 0.026). Employee Happiness Scale scores were significantly higher among nurses working at the Training and Research Hospital (61.72 ± 13.41) than among those working at the State Hospital (52.69 ± 14.51), among unit/ward nurse managers (66.32 ± 12.72) than among ward nurses (57.06 ± 14.41), among those working 0–40 h per week (62.53 ± 15.42) than among those working 41 h or more per week (56.98 ± 14.06).

Bonferroni-adjusted post hoc comparisons indicated a statistically significant difference between among those whose income was equal to their expenses (60.00 ± 10.92) than among those whose income was less than their expenses (58.14 ± 14.95). (mean difference = −6.102, *p* = 0.046, 95% CI [−12.13, −0.07]). However, no statistically significant differences were other groups (*p* = 0.107, *p* = 1.000).

Correlation analysis was performed to determine the relationships between nurses’ age and scale scores. Significant negative correlations were found between participants’ age and their perceptions of sustainable leadership (moderate strength; r = −0.418; *p* < 0.001), between age and their perceptions of informatics leadership (weak strength; r = −0.386; *p* < 0.001), and between age and employee happiness (weak strength; r = −0.168; *p* = 0.035). A strong positive correlation was found between perceived sustainable leadership and perceived informatics leadership (r = 0.767; *p* < 0.001), and a moderate positive correlation was found between perceived sustainable leadership and employee happiness (r = 0.593; *p* < 0.001). A moderate, positive, and statistically significant correlation was found between perceived informatics leadership and employee happiness (r = 0.544; *p* < 0.001) (Table 3).

Both perceived sustainable leadership and perceived informatics leadership were positively and significantly associated with nurses’ employee happiness scores.

Since the Employee Happiness Scale includes a “Managers” domain that may conceptually overlap with the two measures of perceived leadership, a sensitivity analysis was conducted by removing the manager-related items from the scale. The Cronbach’s alpha coefficient for the resulting Employee Happiness Scale, consisting of the remaining four subdimensions, was found to be 0.890. Based on the number of remaining items, the theoretical midpoint was calculated as 51. The participants’ mean score was 49.29 ± 12.22, which was slightly below this midpoint. The correlation analysis showed that the relationships between the two leadership scales and both the overall employee happiness scores and the employee happiness scores calculated after excluding the Managers domain were similar (Table 3).

Regression analysis was performed by including leadership scales individually and together as single variables in the model. The regression analyses demonstrated that both informatics leadership and sustainable leadership were significant positive predictors of employee happiness when examined separately. Informatics leadership explained 29.6% of the variance in employee happiness (β = 0.544, *p* < 0.001), whereas sustainable leadership explained 35.2% (β = 0.593, *p* < 0.001). When both leadership variables were entered simultaneously into the regression model, both remained statistically significant predictors. Sustainable leadership had a standardized coefficient of β = 0.426 (*p* < 0.001), while informatics leadership had a coefficient of β = 0.218 (*p* = 0.030). The combined model was statistically significant (F(2, 155) = 45.729, *p* < 0.001), explaining 37.1% of the variance in employee happiness (R^2^ = 0.371; adjusted R^2^ = 0.363). The reduction in the standardized regression coefficients in the combined model suggests that the two leadership constructs share some explanatory variance. Nevertheless, both retained significant unique associations with employee happiness after controlling for each other.

To assess the potential content overlap between the employee happiness measure and the leadership scales, a sensitivity analysis was conducted by excluding the managers’ domain from the employee happiness scale. The remaining four dimensions were used to calculate an alternative employee happiness score, and the regression analyses were repeated. The results showed that sustainable leadership remained a significant positive predictor of employee happiness (β = 0.314, B = 0.337, 95% CI [0.108, 0.567], *p* = 0.004). Similarly, informatics leadership remained a significant positive predictor (β = 0.224, B = 0.150, 95% CI [0.007, 0.294], *p* = 0.040). The overall regression model was statistically significant (F(2, 155) = 26.772, *p* < 0.001), explaining 25.7% of the variance in the revised employee happiness score (R^2^ = 0.257; adjusted R^2^ = 0.247). Compared with the original regression model (R^2^ = 0.371; adjusted R^2^ = 0.363), the explanatory power decreased after excluding the managers’ domain. The standardized coefficient for sustainable leadership decreased from 0.426 to 0.314, whereas the coefficient for informatics leadership increased slightly from 0.218 to 0.224. Nevertheless, both leadership variables retained their statistically significant positive associations with employee happiness. These findings suggest that the potential content overlap between the managers’ domain and the leadership measures may have contributed to the strength of the original associations. However, the persistence of statistically significant relationships after excluding this domain indicates that the associations were not entirely attributable to this content overlap.

Multiple regression analysis was conducted to evaluate the ability of the Sustainable Leadership Scale, the Informatic Leadership Scale, and the Employee Happiness Scale to explain variations in employee happiness scores by including independent variables that showed statistically significant differences. In the analysis of the Employee Happiness Scale, the independent variables showing significant differences were institution, position, weekly working hours, and perceived income level. The Dummy variable was used for these categorical variables. The variable with the lowest score was considered the reference variable. The coded variables are in Table 4.

**Figure 1:**
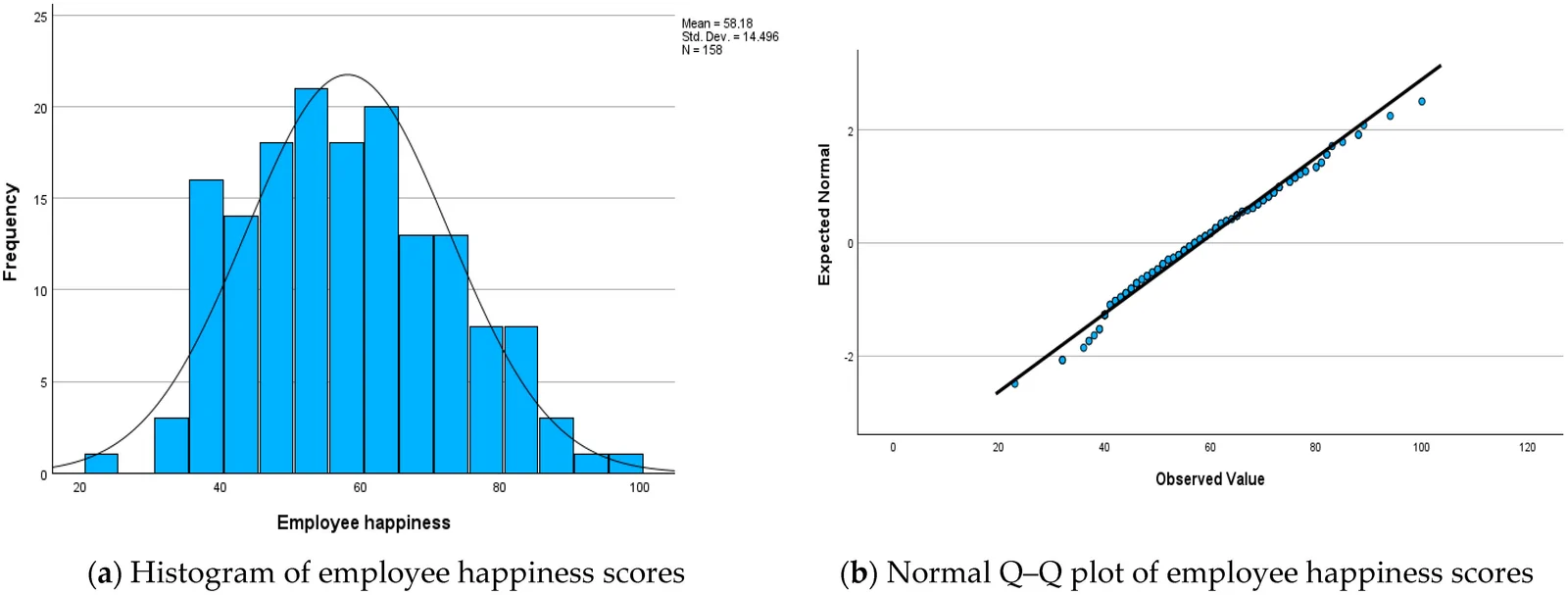
Normal Distribution Plots of Employee Happiness Scale Scores (**a**) Histogram of employee happiness scores and (**b**) Normal Q–Q plot of employee happiness scores. The blue dots in Figure 1b represents the observed data points.

Based on the results, the scale scores had skewness coefficients within the ±1.00 range, suggesting approximately normal distributions. The Kolmogorov–Smirnov test result for the Employee Happiness Scale scores (the dependent variable) was not statistically significant (*p* = 0.200), suggesting no significant departure from normality. The histogram and Q–Q plot are presented in Figure 1.

**Figure 2:**
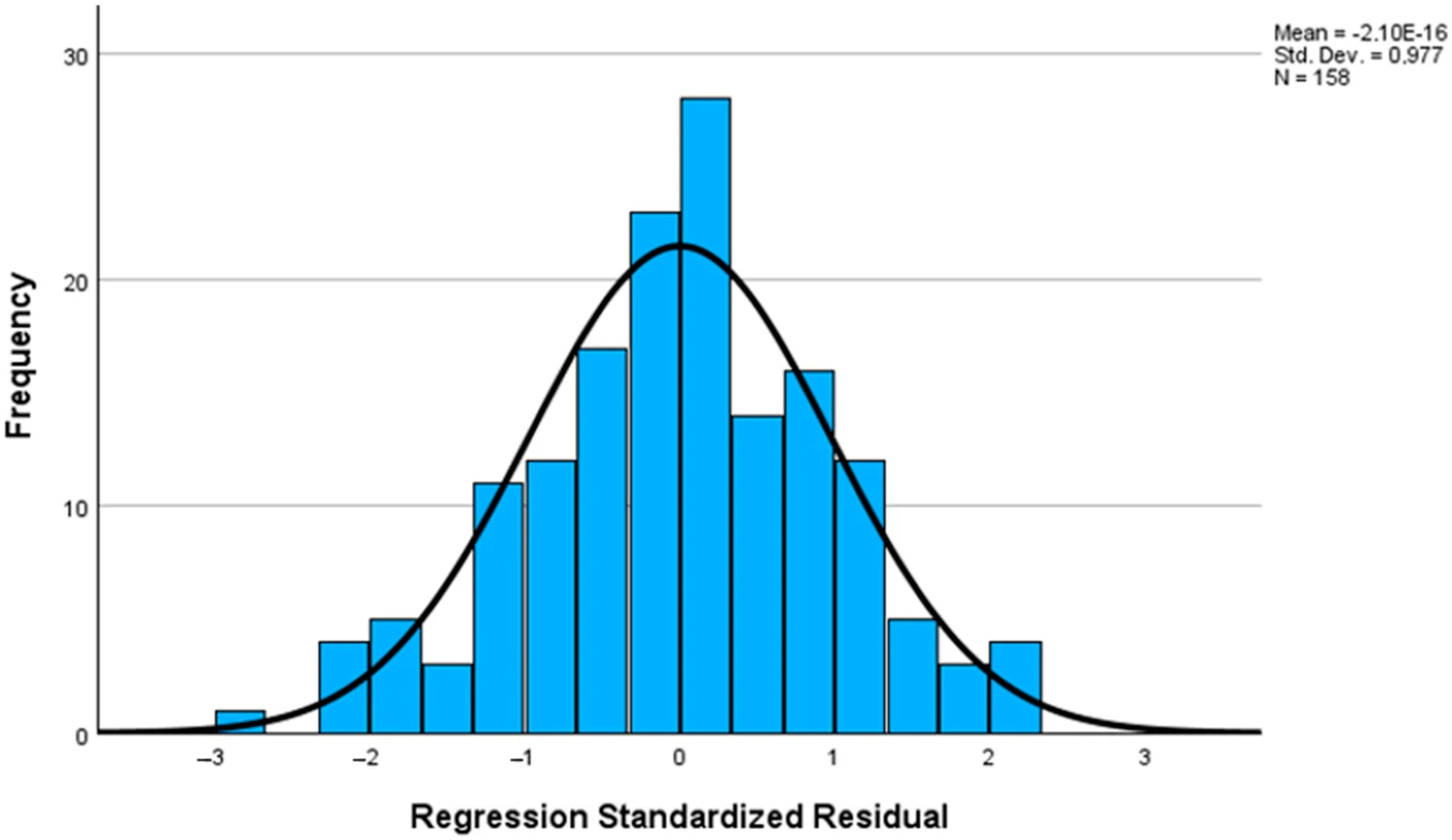
Normal Distribution of Regression Residuals.

**Figure 3:**
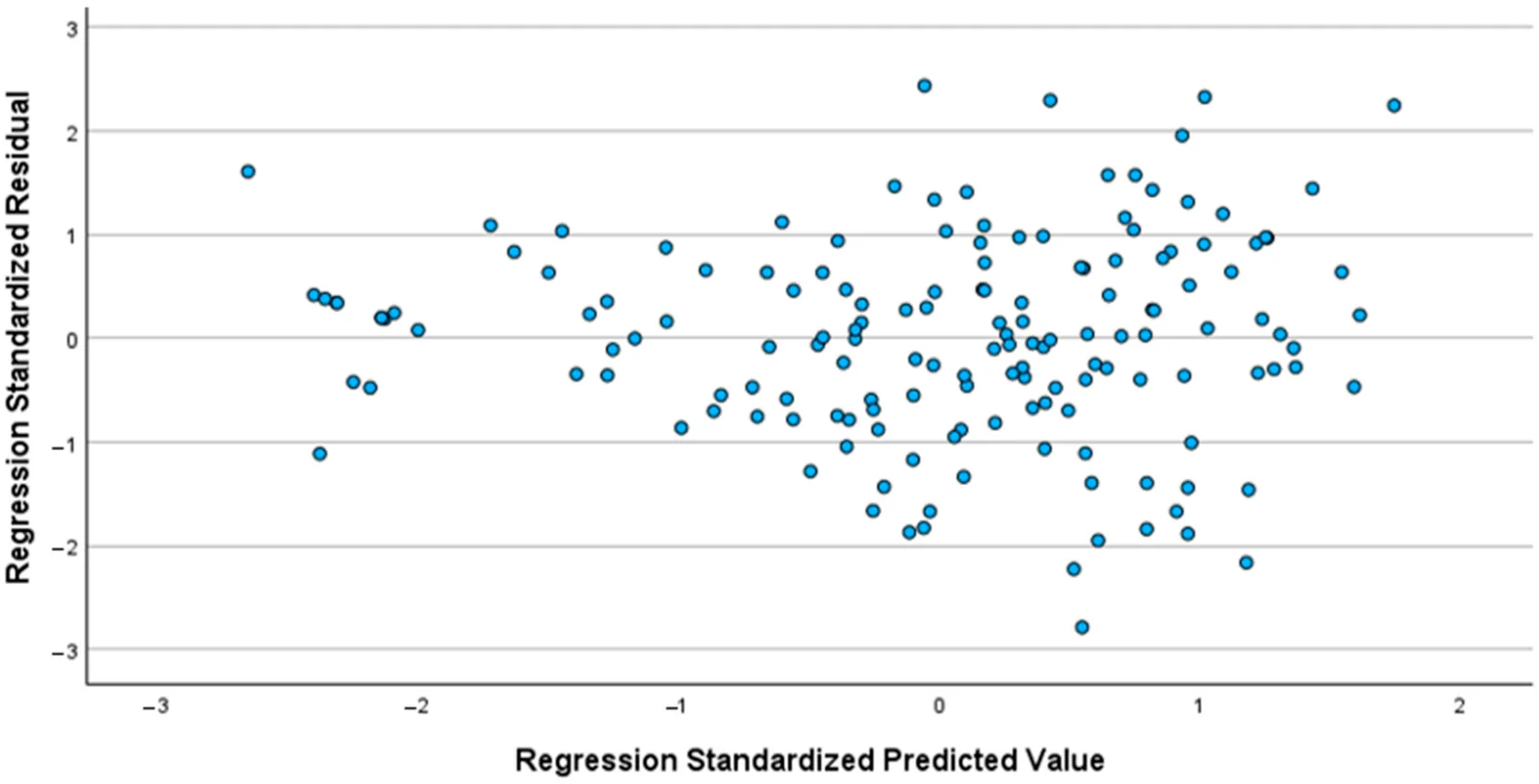
Homoscedasticity of the Variables. The blue dots represent the observed data points.

There were no extreme outliers based on the standardized residuals and Cook’s distance (std. residual min = −2.76, max = 2.31; Cook’s distance max: 0.077); residuals were approximately normally distributed (Figure 2). The residuals showed homoscedasticity, and the errors were independent (Durbin–Watson = 1.650) (Figure 3).

The model explains 41.1% of the total variance in the dependent variable within the analyzed sample. The adjusted R^2^ value, which takes into account the number of variables included in the model, indicates an adjusted explained variance of 38.3%. [(F(7, 150) = 14.949; *p* < 0.001), R^2^ = 0.411, adjusted R^2^ = 0.383]. The Sustainable Leadership Scale and the Informatics Leadership Scale scores were significant positive predictors of employee happiness. A one-standard-deviation increase in the Sustainable Leadership Scale score was associated with a 0.410-standard-deviation increase in the employee happiness score, holding the other variables constant. A one-standard-deviation increase in the Informatics Leadership Scale score was associated with a 0.226-standard-deviation increase in the employee happiness score, holding the other variables constant. Age, employer, position, weekly working hours, and perceived income level were not statistically significant predictors of employee happiness (Table 5).

## 4. Discussion

The findings of this study contribute to understanding the associations between nurses’ perceptions of sustainable leadership and informatics leadership and their levels of employee happiness.

The study found that nurses’ perceptions of sustainable leadership were above the midpoint. Similarly, studies conducted with healthcare workers have indicated that perceptions of sustainable leadership are above the midpoint [5,32]. These findings may reflect nurses’ positive perceptions of employee-centered leadership approaches. This is because sustainable leadership focuses on long-term success and empowers employees [12], while also being associated with greater job satisfaction and feelings of being valued among employees [33].

The literature indicates that nursing informatics leadership is becoming increasingly important for effectively implementing health informatics technologies and ensuring coordination among them [20]. The study found that perceptions of informatics leadership were above the midpoint. This finding may reflect nurses’ positive perceptions of their managers’ roles in using technology and supporting technological transformation in healthcare. However, a qualitative study conducted with nurse managers indicated that they expressed a need for extensive informatics literacy to fulfill the role of informatics leadership [21]. Accordingly, organizing in-service training programs focused on health informatics and technology-based change management could support nurses’ perceptions of informatics leadership and the development of nurse managers’ competencies.

The mean employee happiness score was 58.18 ± 14.49, which was below the cutoff score of 60 specified in the original scale development study. This finding suggests a relatively low average level of employee happiness in the study sample. Similarly, studies have indicated that nurses’ happiness levels are moderate to low [24,34].

While the literature indicates that increased employee happiness is positively associated with the quality of healthcare services [35], it is also known that leadership approaches accepted by employees are associated with employee happiness [26,27,36]. The present study revealed positive correlations between nurses’ perceptions of sustainable and informatics leadership and their employee happiness scores. The regression analysis further indicated that both leadership perceptions were statistically significant predictors of employee happiness within the study model. In interpretation, the possibility of overlap and shared explanatory variance between the two leadership criteria should be considered. Previous studies have shown that sustainable leadership is positively associated with employees’ positive self-esteem and well-being [5,32]. Nevertheless, the observed associations should not be interpreted as evidence of a causal relationship. It is also possible that happier employees evaluate their managers and leadership practices more positively. Furthermore, common response tendencies arising from the use of self-reported measures and unmeasured workplace factors, such as workload, organizational support, and working conditions, may partly explain the observed relationships. Therefore, the findings should be interpreted as associations between nurses’ leadership perceptions and employee happiness rather than as evidence that leadership approaches directly increase employee happiness.

In addition, the study found a negative relationship between age and perceptions of sustainable leadership, perceptions of informatics leadership, and employee happiness. Older participants tended to report lower scores on both leadership perception scales and the Employee Happiness Scale. This finding indicates that younger participants tended to report more positive perceptions of sustainable leadership and informatics leadership. Today, advancements in informatics technology and the growing importance of sustainability are making informatics and sustainable leadership approaches increasingly critical for organizations. In this regard, training, development, and motivation programs could be considered to support employees of different age groups in informatics transformation and sustainability processes.

Within the context of healthcare organizations, the present study contributes to the literature on healthcare management and health informatics by highlighting the associations between nurses’ perceptions of sustainable leadership and informatics leadership and employee happiness. These findings provide an organizational perspective on nurses’ workplace well-being and underscore the relevance of leadership approaches to understanding employee happiness in healthcare settings.

## 5. Conclusions

The study found that nurses’ perceptions of sustainable leadership and informatics leadership were above the theoretical midpoints, whereas their mean employee happiness score was below the cutoff specified in the original scale development study. Older nurses tended to report lower scores on measures of sustainable leadership, informatics leadership, and employee happiness. Furthermore, strong, positive correlations were found between perceptions of sustainable leadership and informatics leadership; positive, significant correlations were also found between both leadership perceptions and employee happiness. These findings indicate that more positive perceptions of sustainable and informatics leadership were associated with higher employee happiness scores.

The multiple regression analysis showed that perceptions of sustainable leadership and informatics leadership were statistically significant predictors of employee happiness in the regression model, which accounted for 38.3% of the variance in employee happiness scores. These findings suggest that perceived sustainable and informatics leadership are relevant correlates of employee happiness in nursing settings.

Healthcare institutions may consider professional development initiatives addressing sustainable and informatics leadership, opportunities for employee participation in decision-making, and organizational practices that facilitate the use of informatics technologies. Nevertheless, the effectiveness of these initiatives in improving employee happiness should be evaluated through longitudinal or intervention studies. Furthermore, further research should consider other individual and organizational factors that may be associated with employee happiness.

### Limitations and Future Research

This study has several limitations. First, the study was conducted in a single province, and the limited number of hospitals in this province may have restricted the population and sample size. Therefore, the findings may not be generalizable to nurses working in different geographical regions or healthcare institutions. Future studies involving larger and more diverse samples from multiple regions and institutions are recommended. Additionally, the voluntary participation of nurses and the relatively low response rate may have introduced selection bias.

Second, the data were collected using self-report questionnaires, which may be subject to response bias and social desirability bias. Furthermore, because nurses’ perceptions of sustainable leadership, informatics leadership, and employee happiness were assessed using the same data collection method, the findings may have been influenced by common method bias. Although the two leadership scales were developed to assess distinct constructs, their high Cronbach’s alpha coefficients indicate internal consistency but do not independently establish construct or discriminant validity. These high values may also reflect potential item redundancy, which was not specifically examined in this study.

Finally, the cross-sectional design reflects participants’ perceptions at a single point in time and does not allow causal relationships to be established between leadership perceptions and employee happiness. Longitudinal studies are recommended to better understand how these relationships may change over time. Moreover, potential confounding factors may have influenced the observed relationships.

Our findings should be evaluated not as evidence of general management effects at the hospital level or inter-hospital differences, but as evidence of the relationships between nurses’ perceptions of individual leadership and employee happiness in the hospitals participating in the study.

## Figures and Tables

**Table 1:** Descriptive data on nurses.

		N	%
Age	$\bar{x}$ ± sd = 33.91 ± 9.15		
Gender	Female	135	85.4
	Male	23	14.6
Employer	Training and Research hospital (TRH)	96	60.8
	State hospital (SH)	62	39.2
Educational status	Associate’s degree	28	17.7
	Bachelor’s degree and higher	130	82.3
Perceived income level	Income less than expenses	58	36.7
	Income equal to expenses	75	47.5
	Income greater than expenses	25	15.8
Marital status	Single	74	46.8
	Married	84	53.2
Position	Unit/Ward Nurse Manager	19	12.0
	Ward nurse	139	88.0
Years of professional experience	0–10 years	96	60.8
	11 years and above	62	39.2
Years of service at the institution	0–10 years	121	76.6
	11 years and over	37	23.4
Weekly working hours	0–40 h	34	21.5
	41 h or more	124	78.5
Work schedule	Daytime only	31	19.6
	On-call and day shifts	113	71.5
	Night shift only	14	8.9

**Table 2:** Nurses’ scale scores for sustainable leadership, informatics leadership, and employee happiness.

	N	Observed Min.	Observed Max.	Mean	SD	95% Confidence Interval	
						Lower	Upper
Sustainable Leadership Scale	158	10	50	35.49	11.37	33.74 ± 10.02	12,602
Informatics Leadership Scale	158	18	90	56.93	18.19		
Employee Happiness Scale	158	23	100	58.18	14.49		

SD: Standard Deviation.

**Table 3:** Pearson correlation analysis between age, sustainable leadership, informatics leadership, and employee happiness scales scores.

		Age	Sustainable Leadership Scale	Informatics Leadership Scale	Employee Happiness Scale	Employee Happiness (Excluding the Manager Subscale)
Age	r 95% CI	1	−0.418 [−0.538–0.279]	−0.386 [−0.511–0.244]	−0.168 [−0.315–0.011]	−0.113 [−0.264–0.044]
	*p*		<0.001	<0.001	0.035	0.158
Sustainable leadership scale	r 95% CI		1	0.767 [0.693–0.824]	0.593 [0.480–0.685]	0.486 [0.355–0.596]
	*p*			<0.001	<0.001	<0.001
Informatics Leadership Scale	r 95% CI			1	0.544 [0.423–0.645]	0.465 [0.332–0.578]
	*p*				<0.001	<0.001

r: Correlation coefficient.

**Table 4:** The coded variables.

Variable	Coding
Employer	State Hospital = Reference Training and Research Hospital = 1
Position	Ward nurses = Reference Unit/ward nurse managers = 1
Weekly working hours,	Those working 41 h or more per week = Reference Those working 0–40 h per week = 1
Perceived income level	Those whose income was less than their expenses = Reference Those whose income was equal to their expenses = 1

**Table 5:** Multiple Linear Regression Analysis of Factors Associated with Employee Happiness.

	B	β	t	*p*	95% CI for B		Correlation Partial	Tolerance	VIF
Constant	19.844		3.054	0.003	7.007	32.681			
Sustainable Leadership Scale	0.523	0.410	3.974	<0.001	0.263	0.783	0.309	0.368	2.715
Informatics Leadership Scale	0.180	0.226	2.291	0.023	0.025	0.335	0.184	0.405	2.472
Age	0.179	0.113	1.488	0.139	−0.059	0.418	0.121	0.679	1.473
Employer	2.219	0.075	1.004	0.317	−2.151	6.589	0.082	0.703	1.422
Position	3.529	0.079	1.105	0.271	−2.782	9.841	0.090	0.760	1.316
Weekly working hours	3.221	0.092	1.321	0.188	−1.595	8.038	0.107	0.817	1.224
Perceived income level	2.077	0.072	1.116	0.266	−1.599	5.753	0.091	0.950	1.053

B: unstandardized B, β: standardized beta coefficients, t: *t*-test statistics.

## Data Availability

The data used in this study were collected from participants with the understanding that they would be used solely for the purposes of this research. Therefore, the data are not publicly available due to confidentiality and ethical restrictions. However, access to de-identified data may be provided by the authors upon reasonable request, subject to applicable ethical and confidentiality requirements and the necessary approvals. The questionnaire codebook and statistical analysis syntax are also available from the corresponding author upon reasonable request, subject to applicable ethical and confidentiality requirements.
